# Fetal outcomes and associated factors of adverse outcomes of pregnancy in southern Chinese women with systemic lupus erythematosus

**DOI:** 10.1371/journal.pone.0176457

**Published:** 2017-04-25

**Authors:** Zhongping Zhan, Ying Yang, Yanfeng Zhan, Dongying Chen, Liuqin Liang, Xiuyan Yang

**Affiliations:** 1 Department of Rheumatology, The First Affiliated Hospital of Sun Yat-sen University, Guangzhou, Guangdong, China; 2 Department of Obstetrics and Gynecology, The First Affiliated Hospital of Sun Yat-sen University, Guangzhou, Guangdong, China; Centro Cardiologico Monzino, ITALY

## Abstract

This study aims to investigate the fetal outcomes and associated factors of adverse pregnancy outcomes (APOs) in pregnant women with systemic lupus erythematosus (SLE). Clinical data from 251 SLE patients with 263 pregnancies from 2001 to 2015 were analyzed retrospectively. APOs occurred in 70.0% of pregnancies, in which pregnancy loss occurred in 28.5%; preterm delivery occurred in 21.3%; intrauterine growth retardation occurred in 12.2%; and fetal distress occurred in 8.0%. Over time, the rate of APOs decreased from 82.8% during 2001~2005 to 59.6% during 2011~2015. In multivariate analysis, predictors of APOs included positive antiphospholipid antibodies (OR 8.4, 95% CI 1.7~40.8, *P* = 0.008), lower complement (OR 3.6, 95% CI 1.3~9.9, *P* = 0.01), hypoalbuminemia (OR 3.2, 95% CI 1.2~8.3, *P* = 0.02), and hypertension (OR 14.6, 95% CI 1.5~141.6, *P* = 0.02). The use of antimalarial medications was associated with lower risk for APOs (OR 0.3, 95% CI 0.1~0.7, *P* = 0.01). In total, 109 patients underwent fetal umbilical artery Doppler in the third trimester. The The adjusted systole/diastole (S/D) ratio, pulsatility index (PI) and resistance index (RI) of SLE patients with APOs were higher than that of patients without APOs (2.9±0.9 vs. 2.4±0.5, *P* = 0.001). Lupus pregnancy was still at high risk of APOs in terms of pregnancy loss and preterm delivery. Umbilical artery Doppler was a good monitor method for APOs in the third trimester.

## Introduction

Systemic lupus erythematosus (SLE) is a chronic autoimmune disease that mainly affects women of reproductive age. Pregnancies in women with SLE resulted in an increase of adverse pregnancy outcomes (APOs) [e.g., stillbirth, premature birth, and intrauterine growth restriction (IUGR)], compared with pregnancies in healthy women[1–3]. Several risk factors of APOs in SLE pregnancies have been identified, such as proteinuria, antiphospholipid syndrome (APS), thrombocytopenia, and anti-SSA/Roantibody[4–6]; but results were varied and controversial. Moreover, previous epidemiological studies demonstrated that Asian SLE patients had higher clinical severity than non-Asian SLE patients[7], while the effects of lupus on the pregnancy outcomes in China still had limited data. In China, fetal Doppler ultrasound examinations were regularly performed during each trimester in all pregnancies. The predictive value of fetal Doppler ultrasound examinations for APOs has been reported. Several studies have demonstrated that abnormal umbilical blood flow was correlated with an increased risk of pre-eclampsia and fetal growth restriction in the general population. The close surveillance could be used to manage the best time of delivery in high-risk patients[8]; while the predictive value of Doppler ultrasound of fetal umbilical artery in lupus pregnancies has not been widely assessed.

We therefore conducted a retrospective study based on a large number of patients during the last 15 years. The objectives of this study were to (1) determine the frequency of APOs in Chinese patients with SLE; (2) explore clinical and laboratory variables that predict APOs; and (3) ascertain the predictive value of Doppler ultrasound of fetal umbilical artery for fetal outcomes in SLE pregnancies.

## Materials and methods

### Study design and patients

We performed a retrospective review of medical records from consecutive SLE patients with pregnancy in the First Affiliated Hospital of Sun Yat-Sen University from 2001 to 2015. Women with SLE fulfilled the 1997 American College of Rheumatology revised criteria for SLE[9]. The outcomes of the pregnancies were systematically checked. Patients with incomplete medical records were excluded. The study was reviewed and approved by Medical Ethical Committee of the First Affiliated Hospital of Sun Yat-San University.

### Data collection

Information was obtained from patients’ recorded data, including demographic data, lupus activity during the first, second, and third trimesters of pregnancy, blood pressure, laboratory data, treatment during pregnancy, and outcomes. Laboratory data included complete blood count, urinalysis, serum albumin, 24-hour proteinuria, complement C_3_ and C_4_, antinuclear antibody (ANA), anti-dsDNA antibody, anti-SSA/Ro, and anti-SSB/La. Tests for antiphospholipid (aPL) antibodies included anticardiolipin antibodies (aCL, aCL-IgG, and aCL-IgM), anti-β2-glycoprotein I antibodies, and lupus anticoagulant. All laboratory tests were performed according to standard methods.

The first trimester of pregnancy was termed before the end of the 12th week of gestation; the second trimester was defined between the 13th and the end of the 27th week; and the third trimester was the last three months of gestation. Hypertension was defined as systolic >140 mmHg and/or diastolic blood pressure >90 mmHg in sitting position in at least two consecutive measurements during pregnancy or the usage of anti-hypertensive drugs. Hypoalbuminemia was defined as serum albumin less than 35g/L. The disease activity of SLE during pregnancy was assessed by SLE pregnancy disease activity index, and active SLE was considered when the scores were equal to or more than 4[10]. The highest index during pregnancy before delivery was considered.

We first defined “lupus relapse” in the method section: lupus relapse was defined as has been suggested in a recent International Consensus[11]: new onset or worsening of specific and associated cutaneous manifestations of SLE, arthritis, one or more hemocytopenias not attributed to immunosuppressive drugs, neurological, cardiopulmonary and renal manifestations, elevated serum creatinine in association with low serum complement and/or elevated titers of anti-dsDNA antibodies.

### Doppler ultrasound examinations of fetal umbilical artery

A fetal Doppler ultrasound examination was performed on all fetuses during the 28th–34th gestational weeks using Voluson E6 (GE Kretztechnik, USA) machine equipped with a 4–8 MHz transabdominal probe. Patients were placed in a semi-recumbent position with a left lateral tilt. Then, using a pulsed wave Doppler on a free loop of the cord, the characteristic sound and shape of the umbilical artery were identified. The Doppler flow parameters of umbilical arteries were recorded, including pulsatility index (PI), resistance index (RI), the peak value of umbilical arteries at end-systole (Vmax, also abbreviate as S) and the peak value of umbilical arteries at end-diastole (Vmin, also abbreviate as D). When the screen showed at least three consecutive wave forms of similar height, the image was frozen, and the Doppler PI of the umbilical artery was estimated. A minimum of three separate readings were averaged before the final values were calculated. S/D ratio (i.e., Vmax/Vmin) was calculated automatically. The values (RI, PI and S/D) was adjusted according to the gestational age at examination. Doppler studies of umbilical artery were avoided during fetal activity and breathing because of the effect of fetal breathing movements on waveform variability.

### APOs

The study-defined APOs included one or more of the following: 1) pregnancy loss, including spontaneous abortion (termination of pregnancy before 20 weeks of gestation caused by natural factors), therapeutic abortion (artificial termination of pregnancy because of life-threatening progression of lupus or obstetric complications), and fetal death (intrauterine fetal demise after 20 weeks of gestation unexplained by chromosomal abnormalities, anatomic malformation, or congenital infection); neonatal death referred to the death of a live infant within 28 days after birth; 2) premature birth (live birth before 37 weeks of gestation); 3) IUGR (birth weight below the 10th percentile of Chinese population according to gestational week at delivery and fetal gender; and 4) fetal distress referred to fetus hypoxia and acidosis, which could endanger the health of the fetus.

### Statistical analysis

Statistical analysis was performed using SPSS 20.0 software. Quantitative variables were described as mean ± standard deviation using *t*-test to compare two groups. Categorical variables were described as frequency and percentage. Two-by-two tables were analyzed by chi-square or Fisher exact test, as appropriate. Factors related to APOs at *P*<0.10 in univariate analyses were entered into a multivariate logistic model. A *P*-value of <0.05 was considered statistically significant.

### Ethics statement

The study was reviewed and approved by Medical Ethical Committee of the First Affiliated Hospital of Sun Yat-San University. Since this is a retrospective study, the Ethical Committee waived that the research could be done based on record review without contacting the patients. Support letter was obtained from the medical director office of the hospital for retrieving retrospective data from the database and records. All the information was kept confidential, and no individual identifiers were collected.

## Results

### Clinical characteristics

Clinical records for 251 SLE patients with 263 pregnancies were reviewed. The 240 women each had one pregnancy, 10 women each had two pregnancies, and 1 had three pregnancies. The sample had a mean age of 28.6±3.9 years at pregnancy. Among the 263 pregnancies, 75 were newly diagnosed with SLE during pregnancy, and 188 were previously diagnosed with SLE, including 149 planned and 39 unplanned pregnancies. The mean disease duration of patients with previously diagnosed SLEwas 69.9±46.8months(3~252months).Of all pregnancies, 141 (53.6%) had active lupus during pregnancy. The most common complication was lupus nephritis, which was identified in 53.6%; followed by thrombocytopenia in 20.9%, heart involvements in 6.5%, lung involvements in 6.1%, and digestive system involvements in 3.0%.Fourteen patients hadantiphospholipid syndrome(14/251, 5.6%), 6 of them received aspirin alone during pregnancy, and 8 were treated with aspirin and low molecular weight heparin.While the treatments of aspirin or heparin could notmodify the outcome of pregnancies.Interms ofimmunosuppressants, there wasno difference betweenpregnancies with orwithout APOs(Table 1).We noted that 52 (52/188, 27.7%) pregnancies were complicated by at least one episode of lupus relapse during their pregnancy. The relapse rate was not different in each trimester.

**Table 1:** Association of different characteristics during pregnancy with APOs: Results of univariate analysis.

Characteristics	Total (n = 263)	With APOs (n = 159)	Without APOs (n = 104)	*P* value
**Medical history** (n, %)				
Prior lupus nephritis history	66 (25.1)	46 (28.9)	20 (19.2)	0.08
Prior APOs history	99 (37.6)	61 (38.4)	38 (36.5)	0.8
**Clinical manifestation** (n, %)				
SLEPDAI>4 during pregnancy	141 (53.6)	112 (70.4)	29 (27.9)	<0.001
Active lupus nephritis	111 (42.2)	100 (62.9)	11 (10.6)	<0.001
Thrombocytopenia	55 (20.9)	42 (26.4)	13 (12.5)	0.007
Leukopenia	16 (6.1)	14 (8.8)	2 (1.9)	0.02
Skin rash	51 (19.4)	36 (22.6)	15 (14.4)	0.1
Joint involvement	31 (11.9)	23 (14.5)	8 (7.7)	0.1
Hypertension	50 (19.0)	44 (27.7)	6 (5.8)	<0.001
**Lab data** (n, %)				
ANA positivity	238 (90.5)	147 (92.5)	91 (87.5)	0.2
Anti-dsDNA antibody positivity	179 (68.1)	118 (74.2)	61 (58.7)	0.008
Anti-Ro antibody positivity	102 (38.8)	73 (45.9)	29 (27.9)	0.003
Anti-La antibody positivity	36 (13.7)	26 (16.4)	10 (9.6)	0.1
Antiphospholipid antibodies positivity	56 (24.9)	51 (38.6)	5 (5.4)	<0.001
Hypoalbuminemia	136 (51.7)	109 (68.6)	27 (26.0)	<0.001
Hypocomplementemia	100 (38.0)	84 (52.8)	16 (15.4)	<0.001
**Medication during pregnancy** (n, %)				
Isodose of prednisone >15mg/d	114 (43.3)	95 (59.7)	19 (18.3)	<0.001
Hydroxychloroquine	87 (33.1)	39 (24.5)	48 (46.2)	<0.001
Aspirin	14(5.3)	10(6.3)	4(3.8)	0.6
Low molecular weight heparin	8(3.0)	7 (4.4)	1(1.0)	0.2
Azathioprine	12(4.6)	8(5.0)	4 (3.8)	0.8
Cyclosporin	5(1.9)	4 (2.5)	1(1.0)	0.7

### Fetal outcomes

A total of 188 live births (71.5%) and 75 pregnancy loss (28.5%) were recorded in the study. One or more APOs occurred in 70.0% (184/263) of patients with SLE. Twenty-five patients (10.0%) had more than one outcome (Table 2). Pregnancy loss and premature births were the most common APOs. Moreover, 12 neonates were diagnosed with neonatal lupus, 9 of which had facial or trunk rash and cardiac involvement, respectively.Among the mothers of 12 neonates with neonatal lupus, 5 of whom were only anti-Ropositiv, and 5 were both anti-Ro andanti-La positive. Six congenital malformations included one cleft lip and palate, one strephenopodia, one polydactyly, two ventricular septal defects, and one atrial septal defect.For those neonates with congenital malformations, all of themothers didnotreceived any teratogenic drugs.In this study, pregnancy induced hypertension (PIH) occurred in 35 cases, of which, 3 cases were gestational hypertension and 32 were preeclampsia. There was no eclampsia occurred.

**Table 2:** Fetal outcomes in pregnant women with SLE.

	N	%
Live birth (n, %)	188	71.5
Full-term birth (n, %)	132	70.2
Premature birth (n, %)	56	21.0
Intrauterine growth retardation (n, %)	32	12.2
Fetal distress (n, %)	21	8.0
Neonatal lupus erythematosus (n, %)	12	4.6
Pregnancy loss (n, %)	75	28.5
Spontaneous abortion (n, %)	18	6.8
Therapeutic abortion (n/N, %)*	48	18.3
Stillbirth (n, %)	7	2.7
Neonatal death (n, %)	2	0.8

* Forty-two for severe SLE complications during pregnancy, including 28 lupus nephritis (LN), 8 multiple involvements (3 with LN and diffuse alveolar hemorrhage, 2 with LN and neuropsychiatric lupus, 1 with LN and lupus meseuteric vaseulitis, and 2 with LN and pulmonary hypertension), 6 severe thrombocytopenia,two for usage of thalidomide before pregnancy, and four for personal reasons.

### Time trends of specific APOs

**Fig 1:**
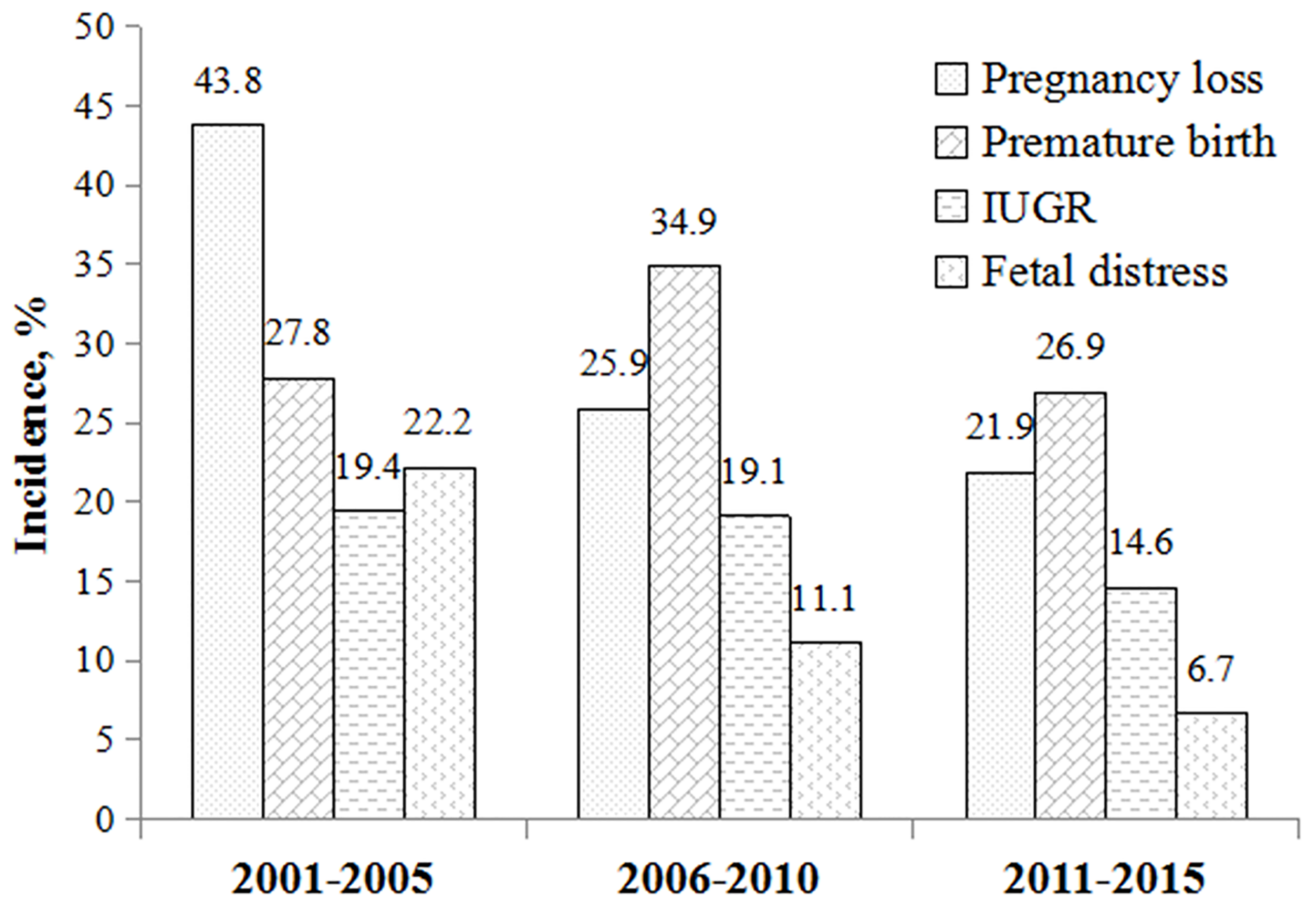
The rate of adverse pregnancy outcomesin women with SLE over the past 15 years.

The constituent ratio of specific APOs had a great change across different time periods. The rate of APOs decreased from 82.8% during 2001~2005 to 59.6% during 2011~2015. Over time, the rate of pregnancy loss decreased, while the proportion of pregnancies resulting in live birth increased. The frequency of fetal distress and IUGR decreased. The rate of premature birth reached its highest point in the second five years and reduced thereafter (Fig 1, Table 3).

**Table 3:** The time trend of APOs in women with SLE over the past 15 years.

Year	Pregnancies N	Live birth N	APOs N(%)	Pregnancy loss N (%)	Premature birth N (%)	IUGR N (%)	Fetal distress N (%)
2001~2005	64	36	53 (82.8)	28 (43.8)	10 (27.8)	7 (19.4)	8 (22.2)
2006~2010	85	63	63 (74.1)	22 (25.9)	22 (34.9)	12 (19.1)	7 (11.1)
2011~2015	114	89	68 (59.6)	25 (21.9)	24 (26.9)	13 (14.6)	6 (6.7)
Total	263	188	184 (70.0)	75 (28.5)	56 (29.8)	32 (17.0)	21 (11.2)

### Clinical features and risk factors of pregnancy loss

In terms of causes of pregnancy loss, therapeutic abortion was predominant, followed by spontaneous abortion, stillbirth, and neonatal death (Table 4). Univariate analysis revealed that nephritis, active lupus, thrombocytopenia, leukopenia, hypocomplementemia, low serum albumin level, aPL positive, ANA positive, and non-use of hydroxychloroquine (HCQ) were associated with pregnancy loss (Table 4). In multivariate analysis, active lupus (OR 12.4, 95% CI = 3.9–39.1, *P*<0.001)and aPL positive (OR 2.9, 95% CI = 1.2–6.8, *P* = 0.01) were independent predictors for pregnancy loss.

**Table 4:** Demographic, laboratory, clinical manifestations of SLE pregnancies with or without pregnancy loss.

Characteristics	Pregnancy loss (n = 75)	Without pregnancy loss (n = 188)	*P* Value
Age of mother (yrs), mean±SD	27.6±4.1	28.3±3.5	0.2
SLEPDAI>4, n (%)	70(93.3)	71 (37.8)	<0.001
Active lupus nephritis, n (%)	55(73.3)	56(29.8)	<0.001
Leukopenia, n (%)	11 (14.7)	5 (2.7)	<0.001
Thrombocytopenia, n (%)	28 (37.3)	27 (14.4)	<0.001
Skin rash, n (%)	18 (24.0)	29 (15.4)	0.1
Hypertension, n (%)	13 (17.3)	37 (19.7)	0.7
ANA positivity, n (%)	73 (97.3)	165 (87.8)	0.02
Anti- dsDNA positivity, n (%)	61 (81.3)	118 (62.8)	0.004
aPL positivity, n (%)	29 (38.8)	27 (14.4)	<0.001
Hypocomplementemia, n (%)	54 (72.0)	46 (24.5)	<0.001
Hypoalbuminemia, n (%)	59 (78.7)	77 (41.0)	<0.001
Isodose of prednisone >15mg/d, n (%)	8 (10.7)	4 (2.1)	0.003
Hydroxychloroquine, n (%)	16 (21.3)	71 (37.8)	0.01

### Clinical features and risk factors of preterm deliveries

Among the 188 live deliveries, 56 (29.8%) delivered preterm. The rates of preterm delivery before and after 34 weeks were 39.3% and 60.7%, respectively. Iatrogenic preterm delivery, preterm premature rupture of membranes, and spontaneous preterm births contributed to 38 (67.9%), 10 (17.9%) and 8 (14.3%) of preterm births, respectively. Two preterm neonatus died with the neonatal mortality rate of 3.6%, both of which occurred in those delivered <34 weeks; one for severe asphyxia with ventricular septal defect, and one for neonatal respiratory distress syndrome. As shown in Table 5, SLE patients with preterm delivery had a higher frequency of active lupus, renal involvement, thrombocytopenia, aPL positive, hypocomplementemia, and hypoalbuminemia, as well as hypertension, than SLE patients with full term delivery. Moreover, patients with preterm delivery received higher rate of >15mg/d prednisone and less rate of hydroxychloroquine. In multivariate analysis, hypertension (OR 5.7, 95% CI 2.4–13.5,*P*<0.001)and hypoalbuminemia (OR 4.1, 95% CI 1.7–9.8, *P* = 0.002) were risk factors for preterm delivery.

**Table 5:** Demographic, laboratory, clinical manifestations of SLE pregnancies with or without preterm deliveries.

Characteristics	Preterm deliveries (n = 56)	Full term deliveries (n = 132)	*P* Value
Age of mother (yrs), mean±SD	28.9±3.9	29.1±3.3	0.7
SLEPDAI>4, n (%)	37 (66.1)	35 (26.5)	<0.001
Active lupus nephritis, n (%)	32 (57.1)	24 (18.2)	<0.001
Thrombocytopenia, n (%)	14 (25.0)	13 (9.8)	0.007
Skin rash, n (%)	10 (17.9)	17 (12.9)	0.4
Hypertension, n (%)	24 (42.9)	15 (11.4)	<0.001
ANApositivity, n (%)	53 (94.6)	113 (85.6)	0.08
Anti- dsDNApositivity, n (%)	40 (71.4)	78 (59.1)	0.1
aPL positivity, n (%)	20 (35.7)	12 (9.1)	<0.001
Hypocomplementemia, n (%)	27 (48.2)	19 (14.4)	<0.001
Hypoalbuminemia, n (%)	47 (83.9)	30 (22.7)	<0.001
Isodose of prednisone >15mg/d, n (%)	29 (51.8)	22 (16.7)	<0.001
Hydroxychloroquine, n (%)	12 (21.4)	53 (40.2)	0.01

### Clinical features and APOs of new onset SLE during pregnancy

Fifty-three patientswith new onset lupus developed APOs (34 with pregnancy loss, 15 with premature, 8 with IUGR, 5 with fetal distress and 5 with neonatal lupus). The main SLEPDAI scores of patients with new-onset SLE was 12.0±8.0. Among the patients who became pregnant after the diagnosis of SLE (also known as “non new-onset lupus”), 106 APOs occurred during pregnancy (41 with pregnancy loss, 41 with premature, 24 with IUGR,16 with fetal distress and 7 with neonatal lupus). The percentage of non new-onset lupuspatients with pre-conception activity was 7.4% (14/188). At the time of conception, 158 cases had prednisone (mean dose 8.5±7.1mg/d, range 2.5~60mg/d), 53 women had hydroxychloroquine, 5 had azathioprine, 1 had cyclosporin and 4 had aspirin.

### Associated factors of APOs

The proportions of active lupus, lupus nephritis, thrombocytopenia, leukopenia, and hypertension were significantly higher in patients with APOs. Compared with patients without APOs, those with APOs had a lower serum albumin and complementary level. Mothers with APOs were more likely to be positive to anti-dsDNA, anti-Ro, and aPL antibodies. Rates of use of prednisone >15mg/d and antimalarial medications also differed between those with or without APO (Table 1). According to multivariate regression analysis, variables that were independently predictive of APOs at any time included aPL antibodies status (OR 8.4, 95% CI 1.7~40.8, *P* = 0.008), lower complement (OR 3.6, 95% CI 1.3~9.9, *P* = 0.01), hypoalbuminemia (OR 3.2, 95% CI 1.2~8.3, *P* = 0.02), and hypertension (OR 14.6, 95% CI 1.5~141.6, *P* = 0.02). Use of antimalarial medications (OR 0.3, 95% CI 0.1~0.7, *P* = 0.01) was associated with lower risk for APOs.

### Fetal umbilical artery Doppler

A total of 109 patients underwent fetal umbilical artery Doppler at 28–34 weeks of gestation. Among these pregnancies, 65 ended in fetal APOs: 20 with preterm delivery, 19 with IUGR, 17 with fetal distress, 7 with stillbirth, and 2 with neonatal death. The examination times of fetus with and without APOs were similar (respectively 31.1±1.6 and 31.7±1.5 gestational weeks, *P* = 0.7). Fetus with APOs had higher S/D ratio, higher PI as well as higher RI compared with fetus without APOs (Table 6).

**Table 6:** Comparison of adjusted fetal umbilical artery Doppler index between patients with and without APOs (mean±SD).

Fetal umbilical artery Doppler	With APOs (n = 65)	Without APOs (n = 44)	*P* value
Adjusted S/D*	1.0±0.3	0.8±0.2	0.001
Adjusted PI*	1.1±0.3	0.9±0.2	0.02
Adjusted RI*	1.3±0.7	0.9±0.1	0.001

*Adjusted values (S/D, RI and PI) according to gestational age = measured values/mean values of the gestational age.

## Discussion

Our study indicated that lupus pregnancy still holds an increase in risk of APOs in terms of pregnancy loss and preterm births, especially in a setting of ethnic race with more disease severity. Studies of APOs in SLE showed significant variation with respective study design and APO definitions. Making direct comparisons between single-center studies was difficult. The similar rate of APOs was observed in a meta-analysis by Smyth *et al*.[12]. In the research, 37 studies with 1,842 patients and 2,751 pregnancies were included. The rates of unsuccessful pregnancy and premature birth rate were 23.4% and 39.4%, respectively. Another multi-center prospective study showed that lower rate of APOs occurred in 22.0% of SLE pregnant patients[13].

Early studies reported that pregnancy with SLE resulted in poor fetal outcomes. In contrast, our study showed that the outcomes of pregnancy with SLE were improving over time. The similar trend was also observed by Moroni *et al*.[14]and Cortés-Hernández *et al*.[15]These improvements may reflect the progress in maternal disease control and the emergence of new therapeutic options. European League Against Rheumatism recommended HCQ to be used during lupus pregnancies since 2007[16]. HCQ has been reported to reduce the risk of lupus flare-up during pregnancy[17], and decrease the rate of neonatal cardiac involvement[18], prematurity, and IUGR[19]. An increased use of HCQ during pregnancy since 2005, from 6.3% in the first five years to 60.9% in the third five years, was also observed in our study, which might be another reason for the improvement of outcomes.

Our study from South China showed that 28.5% of pregnancies ended in pregnancy loss. In studies published after 2000, pregnancy loss ranged between 13% and 35% in mothers with lupus[20–22], which is the most frequent complication of pregnancies in SLE. Therapeutic abortion accounted for more than half of pregnancy loss in our study. SLE activity and aPL increased the rate of pregnancy loss. Previous reports pointed out an increased risk in fetal loss if the mothers had circulating aPL[23, 24]. Besides aPL, SLE activity was also associated with fetal loss[25]. Overall, this data suggested that maintaining inactive disease at and during pregnancy, as well as treating with positive aPL, is important for a successful pregnancy.

Preterm birth was the main cause of perinatal mortality and long-term morbidity. The crude preterm birth rate was 21.3% in our cohort, which was similar to the findings of other studies[26, 27], but higher than the rate estimated in healthy Chinese women (6.2%–7.2%)[28]. In our series, preterm births were frequently related to iatrogenic as option to protect the health of mothers and fetus. Our data indicated that the majority of preterm births occurred between 34 and 37 weeks of gestation. Two neonatal deaths both occurred in those delivered in <34 weeks, so voluntary interruption of pregnancy before this point should be carefully discussed between physicians and the family. Several factors have been reported to be associated with preterm deliveries. However, a consensus has not been established regarding these factors. We found that hypertension and hypoalbuminemia were risk factors for preterm delivery. A systematic review also confirmed the significant link between maternal hypertension during pregnancy and the rate of premature birth[29]. Earlier, Molad *et al*. had reported that adverse live-birth outcomes were significantly associated with lower serum albumin level.

Our data indicated that serologic activity (positive for aPL, low complement level, or hypoalbuminemia) and hypertension were independent risk factors predisposing to APOs. This finding was comparable to those obtained in previous studies[30–32]. As for drug administration, HCQ treatment was associated with lower risk of APOs. Other groups have reported the protective effect of HCQ in SLE pregnancy. Our study highlighted the importance of disease control and the use of HCQ to reduce the onset of APOs. Nonetheless, obtaining optimal outcomes of SLE pregnancy remains a challenge.

In this study, we demonstrated that adjusted three indices (S/D, PI, RI) of the third trimester were relatively higher in those with later APOs. Studies about Doppler ultrasound examination of umbilical arteries in SLE pregnancies are limited. A study from France found that an abnormal umbilical artery waveform on second trimester Doppler examination was the best predictor for APOs in SLE and/or APS[33]. In 77 APS pregnancies, Carmona *et al*. found that fetal outcome correlated with abnormal Doppler examination of umbilical artery at 23–26 weeks[34]. Our results demonstrated that fetal umbilical artery Doppler could be used to stratify the standard of care in pregnancy with SLE; women with abnormal indices of fetal umbilical artery Doppler could start strict monitoring to rapidly identify and treat obstetric complications. However, the findings should be assessed in larger prospective studies.

The study had its own limitations as a retrospective study, such as selection bias, information bias, and small sample size. Given the fetal umbilical artery Doppler, we only performed in the third trimester. The predictive value of fetal umbilical artery Doppler in lupus pregnancies should be also assessed in the first and second trimester.

In conclusion, many pregnant women with SLE may have successful outcomes, but they still remain at an increased risk in terms of pregnancy loss and preterm deliveries. Pregnancy outcomes in our patients have improved during a 15-year period because of the progress of management of SLE and pregnancy. Pregnancy loss remains an important issue, particularly for patients with positive aPL and active lupus activity during pregnancy. Preterm births at <34 weeks of gestation were monitored the most. SLE activity and hypertension influenced pregnancy outcomes. HCQ was safe and prevented APOs of SLE pregnancies. Based from the data from our study, Doppler assessment of umbilical artery in the third trimester was a meaningful monitoring method in pregnant women with lupus.
